# Construction of an immune predictive model and identification of TRIP6 as a prognostic marker and therapeutic target of CRC by integration of single-cell and bulk RNA-seq data

**DOI:** 10.1007/s00262-024-03658-w

**Published:** 2024-03-02

**Authors:** Wenjun Liu, Xitu Luo, Zilang Zhang, Yepeng Chen, Yongliang Dai, Jianzhong Deng, Chengyu Yang, Hao Liu

**Affiliations:** 1 The First Department of General Surgery, The Third Affiliated Hospital of Guangzhou Medical University, Guangzhou, 510150 Guangdong China; 2 Department of Anorectal Surgery, The First People’s Hospital of Foshan, Guangdong, 528010 China; 3 Division of Vascular and Interventional Radiology, Department of General Surgery, Nanfang Hospital, Southern Medical University, Guangzhou, 510000 Guangdong China

**Keywords:** Colorectal cancer, Single-cell RNA sequencing, Prognosis, Immune landscape, TRIP6

## Abstract

**Background:**

Investigations elucidating the complex immunological mechanisms involved in colorectal cancer (CRC) and accurately predicting patient outcomes via bulk RNA-Seq analysis have been notably limited. This study aimed to identify the immune status of CRC patients, construct a prognostic model, and identify prognostic signatures via bulk RNA sequencing (RNA-seq) and single-cell RNA-seq (scRNA-seq).

**Methods:**

The scRNA-seq data of CRC were downloaded from Gene Expression Omnibus (GEO). The UCSC Xena database was used to obtain bulk RNA-seq data. Differentially expressed gene (DEG), functional enrichment, and random forest analyses were conducted in order to identify core genes associated with colorectal cancer (CRC) that were relevant to prognosis. A molecular immune prediction model was developed using logistic regression after screening features using the least absolute shrinkage and selection operator (LASSO). The differences in immune cell infiltration, mutation, chemotherapeutic drug sensitivity, cellular senescence, and communication between patients who were at high and low risk of CRC according to the predictive model were investigated. The prognostic genes that were closely associated with CRC were identified by random survival forest (RSF) analysis. The expression levels and clinical significance of the hub genes were analyzed in vitro. The LoVo cell line was employed to ascertain the biological role of thyroid hormone receptor-interacting protein 6 (TRIP6).

**Results:**

A total of seven main cell subtypes were identified by scRNA-seq analysis. A molecular immune predictive model was constructed based on the risk scores. The risk score was significantly associated with OS, stage, mutation burden, immune cell infiltration, response to immunotherapy, key pathways, and cell–cell communication. The functions of the six hub genes were determined and further utilized to establish a regulatory network. Our findings unequivocally confirmed that TRIP6 upregulation was verified in the CRC samples. After knocking down TRIP6, cell proliferation, migration, and invasion of LoVo cells were inhibited, and apoptosis was promoted.

**Conclusions:**

The molecular predictive model reliably distinguished the immune status of CRC patients. We further revealed that TRIP6 may act as an oncogene in CRC, making it a promising candidate for targeted therapy and as a prognostic marker for CRC.

**Supplementary Information:**

The online version contains supplementary material available at 10.1007/s00262-024-03658-w.

## Introduction

Colorectal cancer (CRC) ranks as the second most common cause of cancer-related mortality globally [1]. Advances in the understanding of CRC pathophysiology have expanded the available therapeutic options, such as immunotherapy [2, 3]. However, the 5-year survival rate of people with CRC metastasis is approximately 14% [4]. Uncontrolled carcinogenic events promote genetic mutations and epigenetic modifications, ultimately resulting in the development of CRC. Genomic instability plays a crucial role in carcinogenesis [5]. Due to molecular heterogeneity, the relapse and mortality rates of CRC might significantly differ among individuals with identical clinicopathological characteristics [6, 7]. Recent genomics advancements have enabled the TCGA Research Network to characterize primary subtypes of CRC [8].

The utilization of single-cell RNA sequencing (scRNA-seq) has shown the potential for investigating the heterogeneity of different malignancies. *Zhang* et al*.* used scRNA-seq to analyze differentially expressed genes (DEGs), distribution, and T cell receptor profiles of T cells from patients with CRC [9]. scRNA-seq facilitates analyses of cellular diversity at the individual cell level and investigations of the functions and disease processes that are associated with distinct cell clusters [10–12]. Via scRNA-seq analysis, *Wang* et al*.* confirmed that monocytes and macrophages significantly influence the tumor microenvironment (TME) of CRC [13]. However, none of these studies were verified via bulk RNA-seq. Bulk RNA-seq provides a comprehensive overview of transcription, although this method has limitations in accurately identifying individual cell types and determining intratumoral heterogeneity [14]. Due to the inconsistencies that have been observed between subtypes that are classified based on a single molecule and clinical outcomes [15–17], combinations of key molecular markers have been proposed to elucidate relationships between molecular events and clinical outcomes and to increase the accuracy of prognosis [18–21]. *Luo* et al*.* used scRNA-seq and bulk RNA-seq to molecularly categorize CRC based on necroptosis and to make prognostic predictions [22]. *Joanito* et al*.* identified two distinct states of epithelial tumor cells and improved the consensus molecular categorization of CRC via scRNA-seq and bulk RNA-seq analyses [23]. In addition, tumor mutational processes continuously influence the somatic genome, resulting in immunodeficiency, aging, and other diseases.

Thyroid hormone receptor-interacting protein 6 (TRIP6) is an adapter protein that utilizes its LIM domain to engage in interactions with numerous proteins [24, 25]. Previous studies have demonstrated that activation of TRIP6 may be induced by the transcriptional activating factor v-rel and acts as a promising target for inhibiting v-rel-induced tumor formation and transcriptional activity [26]. TRIP6, a multifunctional protein, regulates a multitude of biological processes associated with diverse types of cancer; for example, TRIP6 promotes carcinogenesis by enhancing the malignant proliferation and invasion of cancer cells [27, 28]. The overexpression of TRIP6, which is upregulated in glioma cells and tissues, is associated with unfavorable clinical outcomes among glioma patients [29]. However, there is still not enough known about TRIP6, especially what role it plays in CRC.

In this study, we revealed main cell subtypes of CRC that are associated with the surrounding tissues. Furthermore, we established a CRC model that is associated with prognostic genes. By conducting in vitro cell experiments and bioinformatics analyses of data from multiple public databases, we have identified the expression patterns and function of TRIP6 and proposed its potential role as an oncogene in CRC.

## Materials and methods

### Data sources and processing

From the TCGA COAD and READ cohorts, bulk RNA-seq data and clinical information of patients with CRC were downloaded, which included 51 normal colorectal mucosa samples and 647 CRC tissue samples. The scRNA-seq dataset (GSE161277) [30] was obtained from the GEO database (https://www.ncbi.nlm.nih.gov/geo/) and contained data of three paracancerous tissues and eight tumor tissues from patients with CRC. The GSE17536 and GSE39582 [31, 32] datasets (containing data of 177 and 556 patients, respectively, with complete expression profiles and survival information) were also downloaded from the GEO and used as external validation datasets.

### scRNA-seq data analysis

The samples were merged utilizing the R package "Seurat" [33], and cells were isolated by filtration using scRNA-seq with the following exclusion criteria: low expression levels of genes were identified only with nFeature_RNA values > 50 and percent.mt values < 5.

Gene expression levels in isolated cells were standardized using a linear regression model, and the 10 genes with the greatest variability in expression were identified by analysis of variance (ANOVA). The dimensions of the scRNA-seq data were reduced by performing principal component analysis (PCA), and 18 PCs were selected for subsequent analysis using ElbowPlot [34]. The relative location of each cluster was established by conducting t-distributed stochastic neighbor embedding analysis. We annotated the clusters using cell marker genes in the CellMarker database [35] to determine possible positional relationships between cells. We used HumanPrimaryCellAtlasData [36] as a reference for supplementary annotation. Subsequently, we identified marker genes for each cell subtype by analyzing the single-cell expression profiles by adjusting the logfc.threshold argument of the FindAllMarkers function [37] to a value of 1. Finally, genes with a log_2_-fold change > 0.585 and a *P* < 0.05 were selected as unique marker genes of each cell subtype based on our data and on other existing literature [38, 39].

### Construction and validation of a prognostic model

Univariate Cox regression analysis was conducted on the candidate genes in the training set to identify genes that are specifically linked to prognosis. Variables with *P* values < 0.05 were analyzed by LASSO regression, which was conducted using the "glmnet" R package [40] to minimize the number of genes in the risk model. The prognostic model was constructed using the subsequent equation: risk score = gene exp1 × β1 + gene exp2 × β2 + … + gene expression n × βn (gene expression represents the numerical number of the expression levels, and β represents the coefficient obtained via LASSO regression analysis). The R package "survminer" [41] was used to construct survival curves. The R package "survROC" [42] was utilized to generate ROC curves for assessing the risk scores in predicting OS at 1, 3, and 5 years. The prognostic model's validity was validated through internal and external datasets.

### Analysis of immune cell infiltration

The RNA-seq data were processed utilizing the R package “CIBERSORT” [43] to determine the proportions of 22 different infiltrating immune cell types. Associations between risk scores and the presence of tumor-infiltrating immune cells were analyzed using Pearson correlation coefficients.

### Gene set enrichment analysis (GSEA)

GSEA [44] (http://www.broadinstitute.org/gsea) was conducted on all the DEGs in the TCGA dataset using the clusterProfiler package [45]. After performing 1000 permutations, we identified enriched gene sets with a *P* value < 0.05 and a false discovery rate of 0.25. In conclusion, we conducted enrichment analyses using the Kyoto Encyclopedia of Genes and Genomes (KEGG) [46] and Gene Ontology (GO) [47] to illustrate the functional pathways that differentiate high-risk and low-risk groups.

### Gene set-variant analysis (GSVA)

GSVA [48] was utilized to assess gene set enrichment in the transcriptome data. The gene sets were acquired from the Molecular Signatures Database (MSigDB) [49] (http://www.gsea-msigdb.org/gsea/index.jsp), and limma software was utilized to perform GSVA, which can accurately assess possible differences in biological function across various samples.

### Analysis of chemotherapy sensitivity

We predicted the sensitivity of each CRC sample to chemotherapy using the Genomics of Drug Sensitivity in Cancer (GDSC) database [50] (https://www.cancerrxgene.org/) and the R package “pRRophetic” [51]. We screened drugs using Spearman’s analysis to evaluate correlations between the IC_50_ values and risk scores of various medicines.

### Tumor mutational burden (TMB) analysis

We analyzed TCGA mutation annotation data using the "Maftool" R package [52]. The difference in TMB between the two risk groups was assessed. In addition, waterfall plots were generated to visualize mutations in the 30 genes that were most frequently different between the low- and high-risk groups. After performing SubMap analysis, the results were visualized with the “complexHeatmap” R package [53].

### Cell communication analysis

The CellPhoneDB database [54] (version 4.0) was used to examine ligand‒receptor interactions by analyzing the single-cell expression patterns of relevant molecules. Additionally, separate analyses of differences in cell communication between the high-risk and low-risk groups were conducted.

### Random survival forest (RSF) analysis

The RSF algorithm was applied to assess the significance of associated genes via the R software “randomForestSRC” [55]. This algorithm involved 1000 iterations with Monte Carlo simulation (*n* rep = 1000). Genes that had a relative relevance greater than 0.3 were selected for inclusion in the final signature.

### Clinical specimens and immunohistochemistry (IHC)

The harvested specimens were stored in a solution specifically designed for preserving tissues (Miltenyi Biotec, catalog number 130–100-008) from February 2020 to February 2023 from CRC patients in the First People's Hospital of Foshan. All the specimens were cut into pieces that were approximately 1 mm × 1 mm in size and incubated in preheated digestion solution with an enzyme mixture (Sigma‒Aldrich) at 37 °C for 30 min. The tissue samples were fixed, paraffin-embedded, dewaxed, rehydrated, and subjected to antigen retrieval. The samples were stained with primary antibodies at 4 °C overnight and then incubated with a suitable secondary antibody for 30 min at 37 °C. Next, the samples were visualized using DAB solution. Finally, images were captured using an optical microscope. Quantitative analysis was performed on five representative images at a magnification of 40 × using ImageJ software.

### Cell culture and transfection

The NCM460, LoVo, HCT-116, and SW620 cell lines were acquired from the Shanghai Cell Bank of the Chinese Academy of Sciences (Shanghai, China). The cells were cultivated in DMEM medium with 10% FBS (Life Technologies, USA) and penicillin (100 U/mL) and streptomycin (100 U/mL) in a humidified environment at 37 °C with 5% CO_2_. A scrambled shRNA and TRIP6 shRNA were purchased from GeneChem (Shanghai, China). The transfections were performed using Lipofectamine 3000 (Invitrogen, Carlsbad, CA) according to the manufacturer’s instructions [56].

### Western blotting analysis

Western blotting assays were conducted using a previously published protocol [57]. Total proteins were extracted from CRC samples with RIPA buffer (Thermo Fisher Scientific, USA). The protein concentrations were measured with a BCA assay kit (Thermo Fisher Scientific, USA). An equivalent amount of protein from each sample was separated using SDS‒PAGE. Then, the proteins were transferred to polyvinylidene membranes (Millipore; Burlington, MA, USA). The membranes were blocked with Tris-buffered saline-0.1% Tween-20 (TTBS) supplemented with 5% skim milk for 2 h at 25 °C. The membranes were then incubated at 4 °C overnight with the following primary antibodies at the indicated dilutions: anti-CYP2W1 (1:500, PA5-101315, Invitrogen), anti-GDE1 (1:1000, PA5-43012, Invitrogen), anti-PTPN6 (1:1000, ab124942, Abcam), anti-PTTG1IP (1:500, ab128040, Abcam), anti-SEC61G (1:500, PA5-21384, Invitrogen), and anti-TRIP6 (1:500, ab137478, Abcam) antibodies. Next, the membranes were incubated with a secondary antibody, anti-rabbit IgG-horseradish peroxidase conjugate (1:4000; Cell Signaling Technology) for 1 h at 25 °C. The expression levels of the target proteins were detected using an ECL kit (Bio-Rad, USA) and analyzed via ImageJ software ((NIH V1.8.0.112, USA).

### Reverse transcription-qPCR (RT-qPCR) analysis

TRIzol reagent (Roche) was used to extract total RNA from cultivated cells. Next, reverse transcription was performed on each sample using the PrimeScript™ RT Reagent Kit with gDNA Eraser (Takara). Gene expression was quantified with TB Green® Premix Ex Taq™ II. The primer sequences that were utilized are provided in Table S1. Relative gene expression levels were determined utilizing the comparative 2^−ΔΔCT^ method [58].

### CCK-8 and Colony-formation assays

The cells were evenly distributed in 96-well plates. After a 96-h incubation period, the viability of cells was assessed according to the manufacturer’s instructions using a CCK-8 assay. For colony formation assays, a total of 1 × 10^3^ LoVo cells were incubated in 6-well plates for 2 weeks. Subsequently, the cells were immobilized using a 4% paraformaldehyde (PFA) solution for 15 min, followed by staining with 0.2% crystal violet for 15 min. The images were acquired with a digital camera and processed using ImageJ software.

### Wound-healing and Transwell assays

The cell monolayer was linearly wounded using a pipette tip, and cells were cultured in serum-free medium using a pipette tip in wound-healing assays. The wound-healing rate was imaged and examined after 48 h. In a 24-well transwell cell culture apparatus containing 8-μm pore size multiporous polycarbonate membrane inserts, migration assays were performed. Briefly, the upper chamber was filled with a suspension of cells, while the bottom chamber was filled with medium that included 10% FBS. The filters were removed, rinsed twice with PBS, fixed with methanol, and stained with 0.5% crystal violet reagent following a 24-h incubation at 37°C in 5% CO_2_. The cells located on the upper side of the filter were removed using cotton swabs. In order to identify migrated cells on the lower side of the filter, particular cross-sectional fields on the filters were quantified. Transwell invasion assays were conducted using Matrigel-coated transwells under the same conditions.

### Flow cytometry assay

LoVo cells were treated with prechilled 70% ethanol for 12 h, incubated with RNase in a 37 °C water bath for 30 min, and prepared with a propidium iodide (PI) detection kit (KeyGen, China). The cell nuclear DNA was labeled with PI at 4 °C. The cells were examined using flow cytometry within 1 h following the manufacturer's recommendations to analyze cell cycle distribution. To analyze apoptosis, the LoVo cells were trypsinized and collected by centrifugation at 500 × g for 3 min at 4 °C. Afterward, the cells were suspended again using a FITC Annexin V Apoptosis Detection Kit (BD Biosciences). The data were analyzed using ModFit-LT software (Verity Software, Topsham, ME) and FlowJo V10 software (Treestar, Inc. Ashland OR).

### 5‐Ethynyl‐2′‐deoxyuridine (EdU) assay

After transfection, LoVo cells were seeded in 12-well plates with 14 µl of slippers and cultivated with 50 µm of EdU reagent (diluted 1:1000 in DMEM with 10% FBS) for 2 h at 37 °C. The cells then were fixed with 4% paraformaldehyde (PFA) and stained with Hoechst solution.

## Statistical analysis

The data analyses were performed using GraphPad Prism 8.0 and R software (v.4.1.1). The data are displayed as the average value plus or minus the standard deviation (SD). Correlation coefficients, namely Pearson and Spearman, were computed to assess relationships between variables. To evaluate the differences between two groups, the Wilcoxon rank-sum test or a two-tailed unpaired t-test was utilized. It was determined that differences between two categories were statistically significant when *P < 0.05, **P < 0.01, and ***P < 0.001.

## Results

### Identification of CRC cell subtypes

**Fig. 1:**
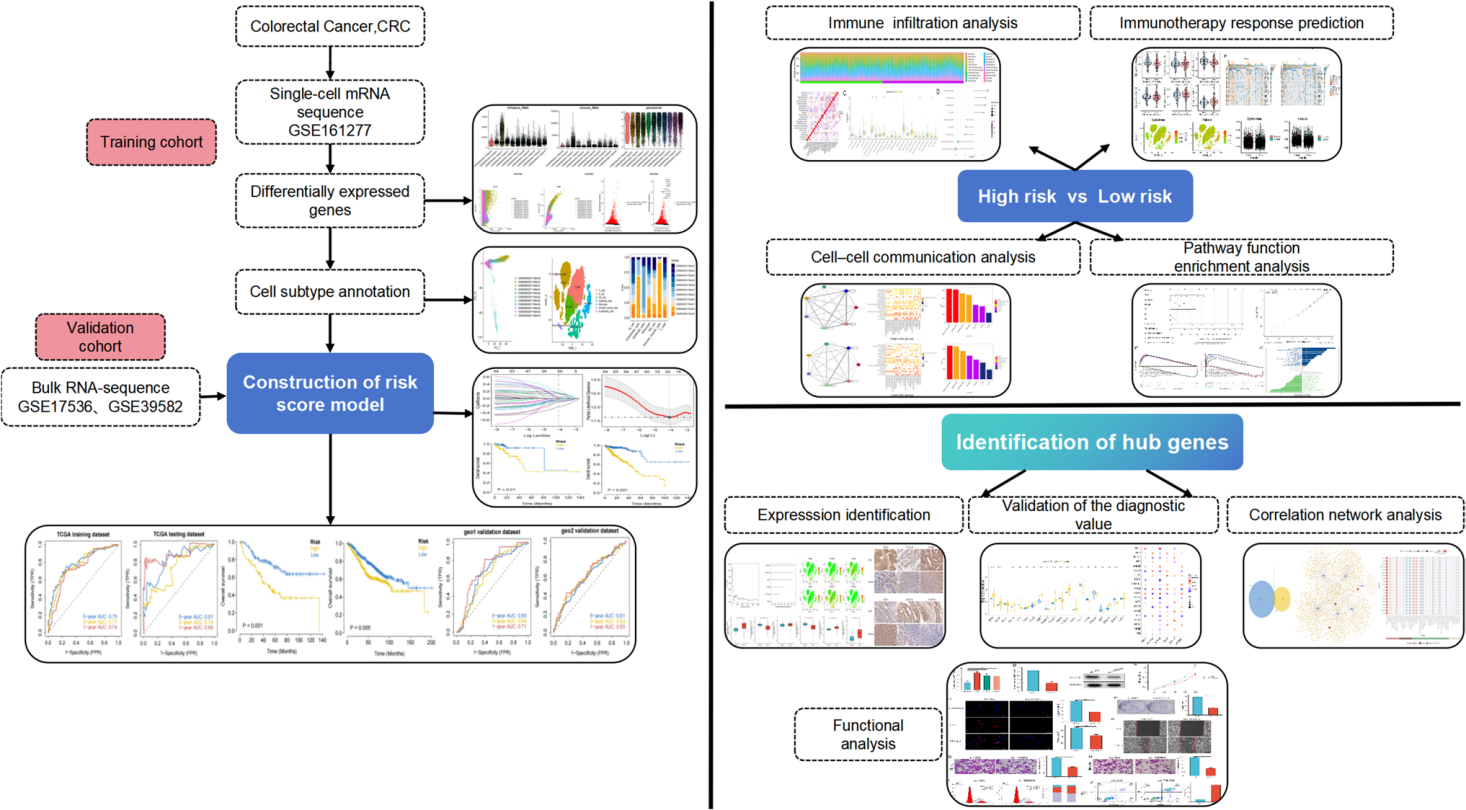
Flowchart of the study

**Fig. 2:**
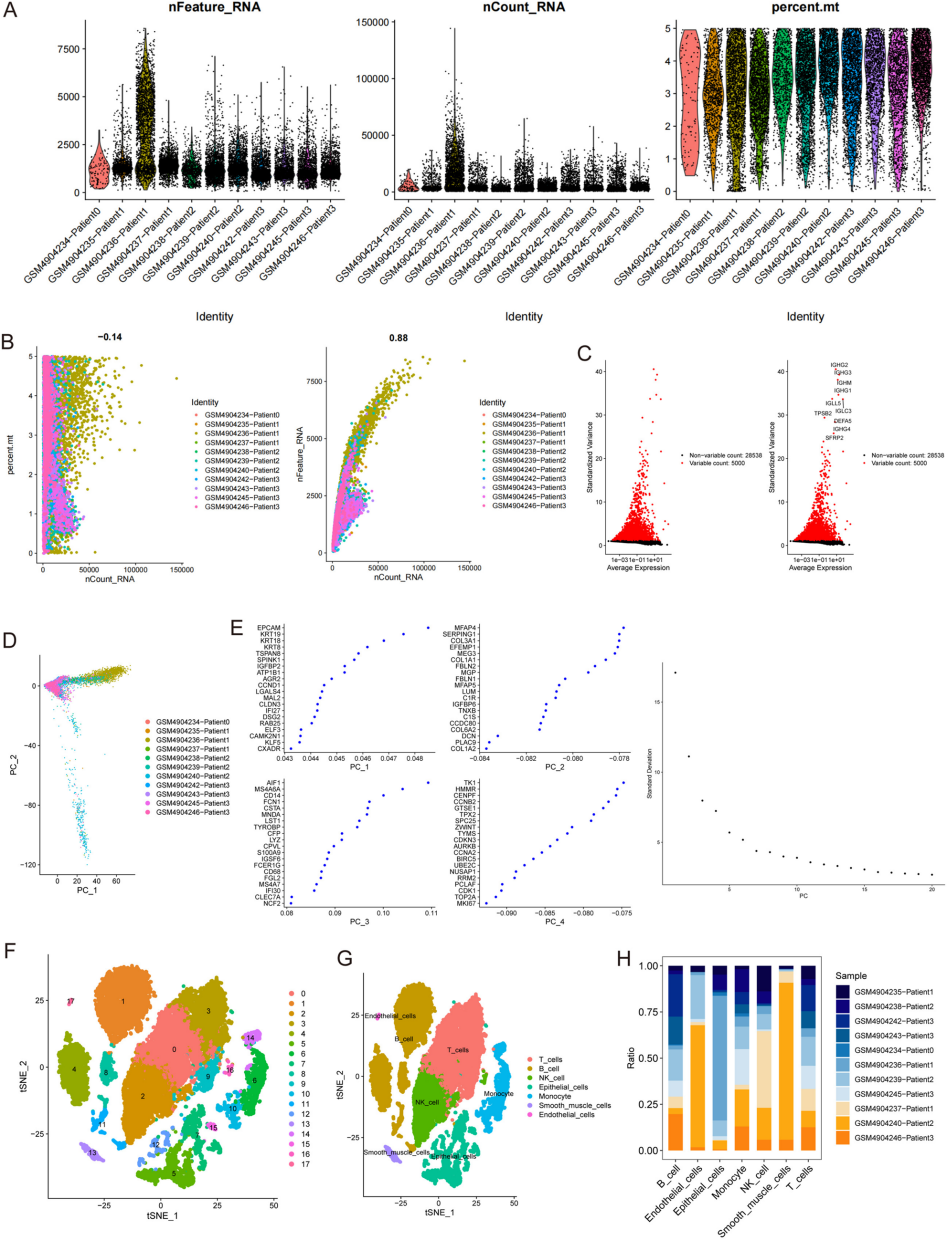
scRNA-seq data revealed seven cell clusters with diverse annotations and heterogeneous expression patterns in CRC. **A, B** A total of 18,367 cells were identified. **C** Diagram showing variations in gene expression levels in all CRC cells. The red dots represent genes with highly variable expression, and the black dots represent genes with stable expression. **D** PCA clearly separated individual CRC cells. **E** The top 20 PCs at *P* < 0.05, as determined via PCA. **F** A total of 18 clusters were identified by dimension reduction and clustering.** G** The 8 clusters were identified by their respective marker genes as different types of cells. **H** Proportions of different cell types based on an analysis of bulk RNA-seq data from normal and CRC samples

The research methodology that was employed in this study is visually represented through the graphical flowchart (Fig. 1). First, we examined 18,367 cells for further analysis based on the parameter settings (nFeature_RNA > 50 and percent.mt < 5; Fig. 2A, B). Second, we identified the top approximately 5000 highly variable genes and focused on the 10 DEGs with the greatest variation in expression in the isolated cells by using ANOVA (Fig. 2C). Eleven single-cell samples were scattered and showed a logically distributed pattern according to PCA (Fig. 2D). PCA and dimension-reduction analysis of the 20 genes revealed varying scores across multiple dimensions, and 18 principal components (PCs) were selected for subsequent analysis (Fig. 2E). The cells formed 18 clusters in descending orders (Fig. 2F). The various groups were labeled by identifying marker genes, which revealed seven cell clusters (B cells, endothelial cells, T cells, monocytes, natural killer (NK) cells, smooth muscle cells, and epithelial cells; Fig. 2G). The composition of cell subclusters in each tumor sample varied considerably (*P* < 0.01; Fig. 2H). Among the cells in the tumor tissues, the epithelial cell subtype was present in the highest proportion. We selected 902 epithelial cell marker genes for subsequent analysis.

Construction and validation of the predictive model based on the marker genes of the largest cell subtype.

**Fig. 3:**
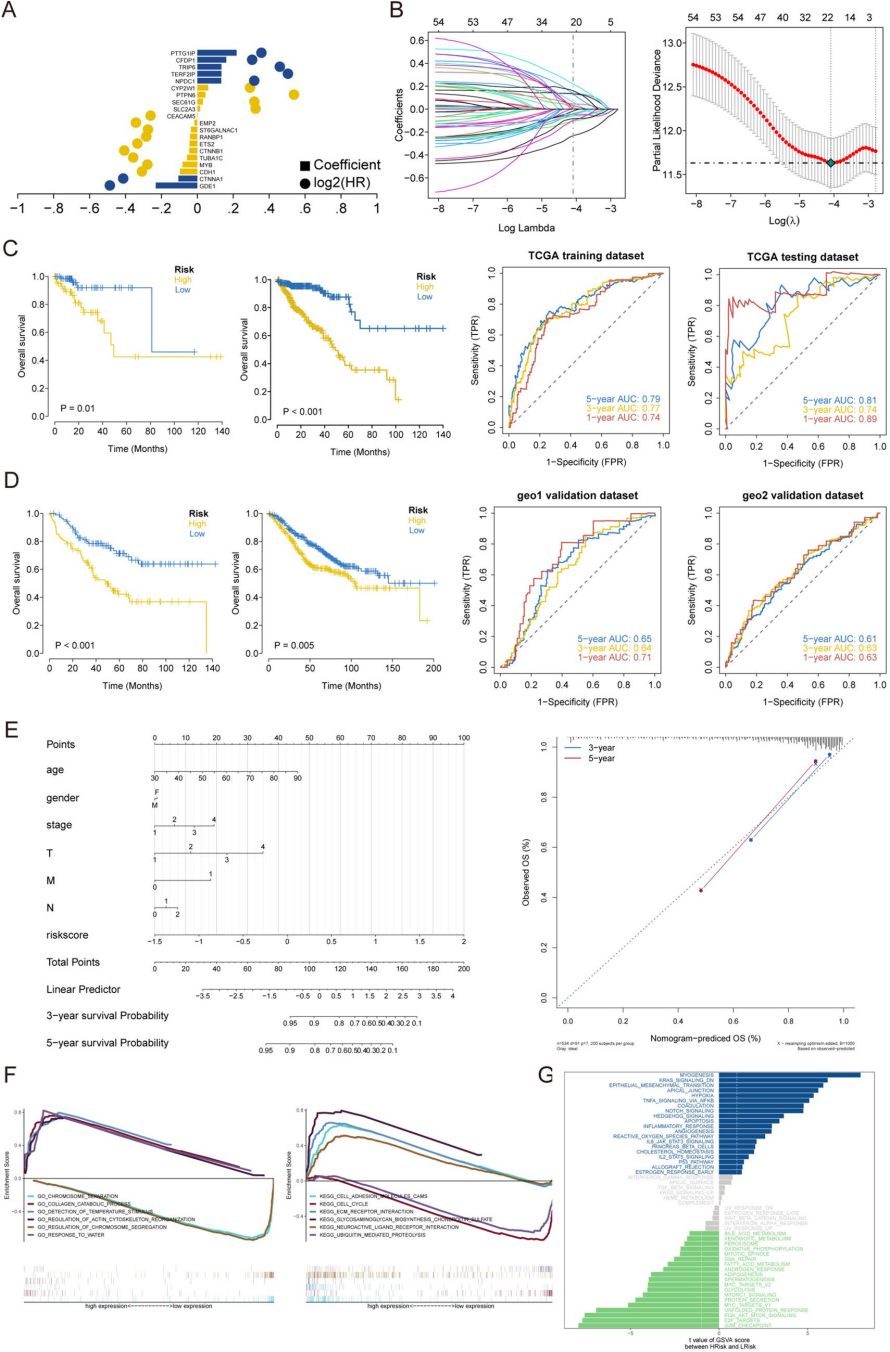
Construction and validation of the prognostic model with TCGA cohort data.** A** Univariate Cox regression analysis of OS-related genes. **B** LASSO regression of OS-related genes. **C, D** K‒M and ROC curve results. **E** Construction of a nomogram and analysis of key biological characteristics. **F** GSEA of enriched GO and KEGG terms between the high- and low-risk groups. **G** GSVA of all genes in the high- and low-risk groups was conducted to identify enriched pathways

Univariate Cox regression was performed to analyze the TCGA training cohort. We identified 20 genes that were significantly associated with OS (Fig. 3A). To measure the risk associated with OS, a standardized risk score was generated for each individual sample by integrating the LASSO coefficients (Li) and the expression levels of the RNAs (Expi) to construct a model. The results are displayed in Fig. 3B. Patients were classified into low-risk and high-risk groups based on the median risk score. Kaplan–Meier (KM) analysis demonstrated that individuals with high risk scores had shorter OS than did those with low risk scores (Fig. 3C). The AUC values of the ROC curve for OS at 1, 3, and 5 years exceeded 0.74, suggesting that the risk model demonstrated strong performance. Based on validation via internal and external validation sets, KM analysis revealed that individuals with high risk scores had substantially shorter OS compared to those with low risk scores (Fig. 3D), and the ROC curves (AUC values) for OS at 1, 3, and 5 years exceeded 0.61.

### Nomogram construction and functional enrichment analysis

To identify independent predictive markers, we analyzed risk scores and clinical parameters, such as age, sex, M stage, T stage, N stage and OS, by univariate and multivariate Cox analyses. The outcomes are presented in the nomograms that incorporate many independent prognostic factors (Fig. 3E). The results suggest that the risk score is highly predictive of the OS of CRC patients and functions as an independent prognostic factor. The related pathways that had the highest degree of enrichment between the high- and low-risk groups were identified via GSEA; in the high-risk group, these pathways included “collagen catabolic process” and “detection of temperature stimulus”. Furthermore, the high-risk group exhibited an enrichment of pathways associated with cell-adhesion molecules and extracellular matrix–receptor interactions, whereas the low-risk group displayed an enrichment of pathways related to phagosomes (Fig. 3F). The GSVA results showed that myogenesis, apical junction, the KRAS signaling pathway, and EMT were uniquely enriched in the high-risk group, and the G2M checkpoint, E2F targets, and PI3K/Akt/MTOR pathways were enriched in the low-risk group (Fig. 3G).

Analysis of immune cell infiltration in the high- and low-risk groups.

**Fig. 4:**
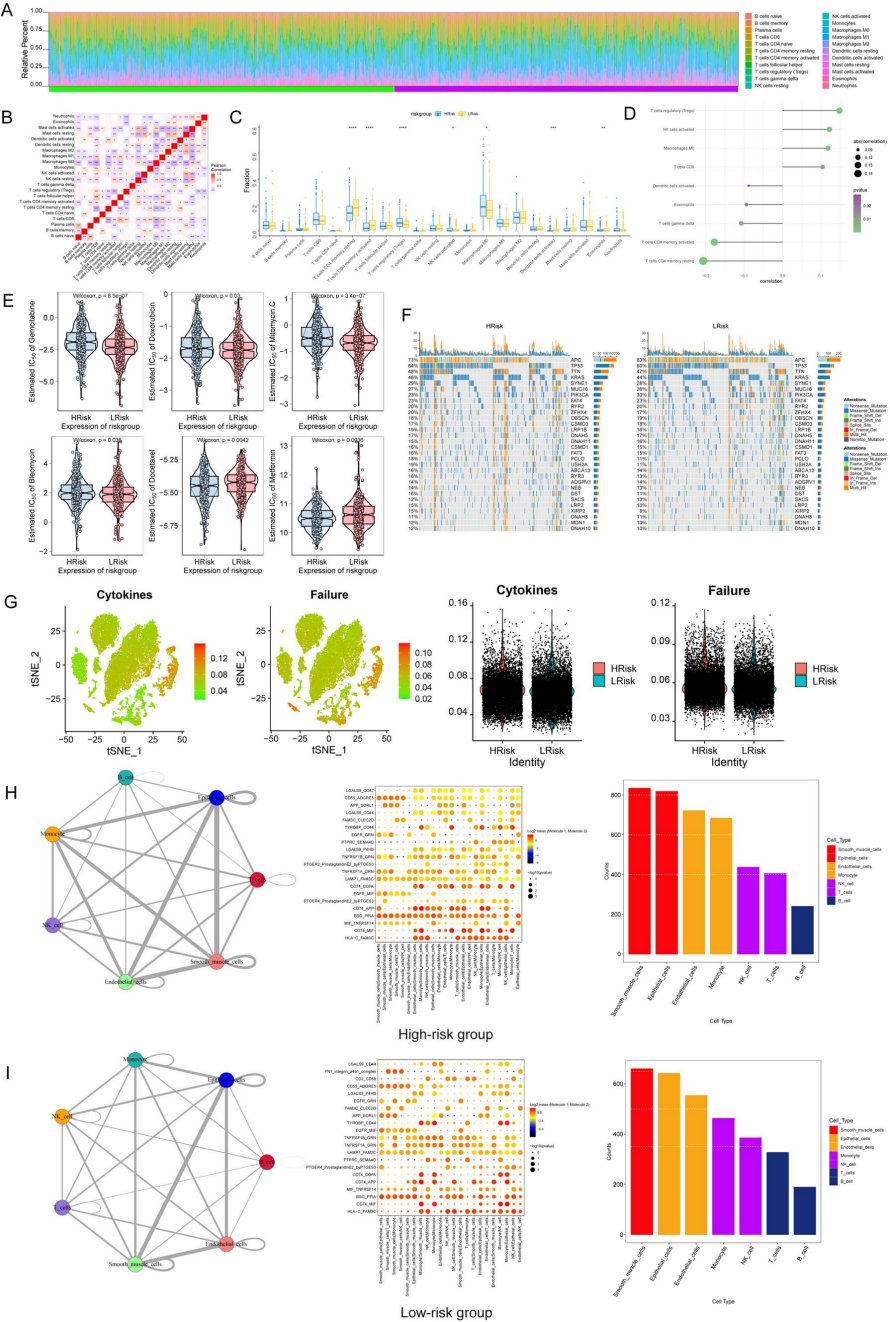
Immune status based on the prognosis-related risk score. **A** Histogram showing the percentages of immune cells in each sample. **B** Correlations among the infiltration of different types of immune cells. **C** Proportions of different types of infiltrating immune cells in the high- and low-risk groups. **D** Univariate Cox analysis of risk scores for different types of immune cells. *P < 0.05, **P < 0.01, ***P < 0.001; ns, not significant. **E** Analysis of the sensitivity to common chemotherapeutic drugs of patients at high and low risk. **F** TMB in the high- and low-risk groups was predicted using risk models. **G** Expression and distribution of senescence- and cytokine-related genes in each cell. **H, I** Cell–cell interactions among different cell types involved in CRC

The distributions of different immune factors were inconsistent across samples (Fig. 4A), and there were marked correlations among the immune factors (Fig. 4B).The low-risk group had elevated numbers of resting memory CD4 T cells, active memory CD4 T cells, activated dendritic cells, and eosinophils, and the levels of infiltrating regulatory T cells, activated NK cells, and M0 macrophages were decreased (Fig. 4C). Furthermore, there were substantial positive correlations identified between the risk score and the proportions of Tregs and activated NK cells, and significant negative correlations were observed between the risk score and the proportions of resting and activated memory CD4 T cells (Fig. 4D).

### Evaluation of potential of immunotherapy in CRC

A strong correlation existed between the risk score and chemotherapy drug sensitivity such as gemcitabine, doxorubicin, mitomycin C, bleomycin, docetaxel, and metformin (Fig. 4E). Furthermore, the high-risk group had a greater prevalence of mutations compared to the low-risk group (Fig. 4F). Furthermore, the levels of senescence and cytokine scores were markedly elevated in the high-risk group compared to the low-risk group (Fig. 4G).

### Cell‒cell communication

High binding interaction scores of HLAC with FAM3C, CD74 with MIF, and BSG with PPIA were observed in multiple cell types both in high-risk and low-risk groups. The number of ligand–receptor gene pairs associated with each cell group was quantified. Epithelial cells and smooth muscle cells exhibited significant potential to interact with different types of cells (Fig. 4H, I).

### Predictive gene validation in CRC

**Fig. 5:**
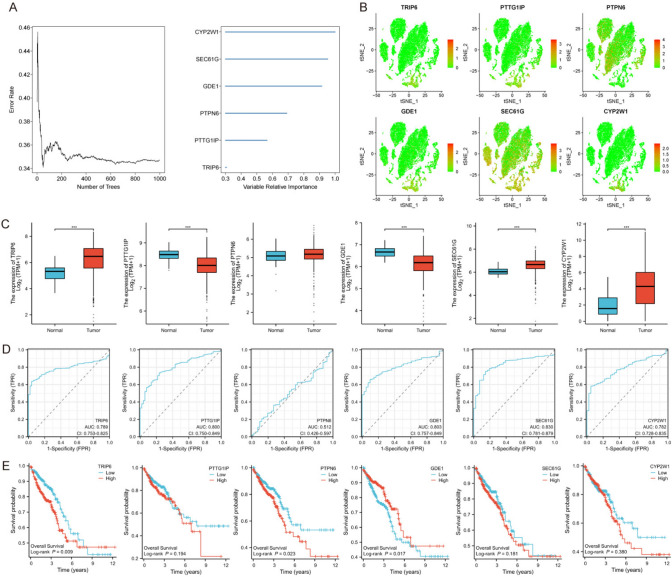
Identification of hub genes in CRC.** A** RSF and expression-level analyses of prognosis-related genes. **B** Expression levels and distribution of *TRIP6, PTTG1IP, PTPN6, GDE1, SEC61G*, and *CYP2W1* in different tumor subtypes. **C** Expression levels of 6 DEGs between CRC tissues and normal tissues. **D** ROC curves representing disease predictions according to six key genes. **E** K‒M curves showing OS of patients with CRC grouped according to the expression levels of key genes

RSF analysis was performed for 20 modeled genes. Six genes met the relative importance criterion of 3% or more and were used as the final marker genes (Fig. 5A). Expression of these six genes in different tumor subtypes was analyzed (Fig. 5B). The expression levels of *TRIP6*, *SEC61G*, and *CYP2W1* were upregulated, whereas those of *PTTG1IP* and *GDE1* were decreased in the CRC group compared with the normal tissue group (Fig. 5C). *TRIP6, PTTG1IP, GDE1, SEC61G,* and *CYP2W1* showed good performance in predicting patient outcome (Fig. 5D). KM plot analysis revealed a strong positive associations between GDE1 expression and overall survival, whereas the expression levels of *PTPN6* and *TRIP6* were negatively correlated with overall survival (Fig. 5E).

### Analysis of regulatory networks

**Fig. 6:**
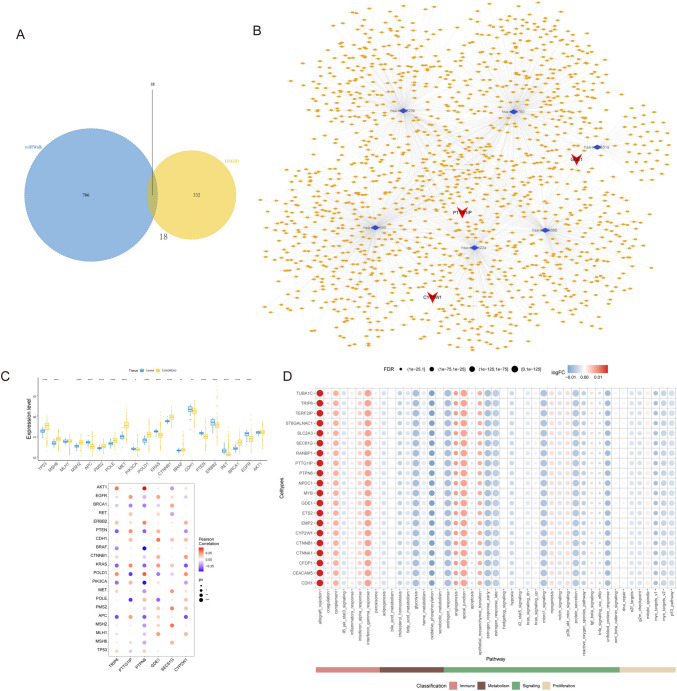
Prediction of the regulatory network of hub genes. **A** Associations of miRNAs with human CRC progression and six hub genes based on the HMDD and the miRWalk database. **B** Visualization of the ceRNA network of the six hub genes. **C** Correlations between the six-gene signature and 20 genes related to CRC. **D** Quantification of the hallmark pathway levels of the hub genes in single cells

First, we identified mRNA–miRNA relationship pairs for these six key genes using the miRWalk database, obtaining 784 miRNAs in total. Subsequently, we retained only the mRNA–miRNA relationship pairs that involved CRC-associated miRNAs, obtaining four mRNAs and 18 miRNAs (Fig. 6A). We subsequently identified 1,879 interaction relationship pairs, including six miRNAs and 1,430 lncRNAs. Finally, a ceRNA network was constructed using Cytoscape (v3.7; Fig. 6B). An analysis was performed on the expression levels of the 20 genes that had the highest correlation scores based on 9119 genes that are related to CRC using the GeneCards database (https://www.genecards.org/). The expression levels of these 20 genes correlated with those of the six hub genes (Fig. 6C). The correlations between key genes and hallmark pathways were determined, and the findings demonstrated a significant positive correlation between the pivotal genes and EMT (Fig. 6D).

### Verification of prognosis-related hub genes in CRC

**Fig. 7:**
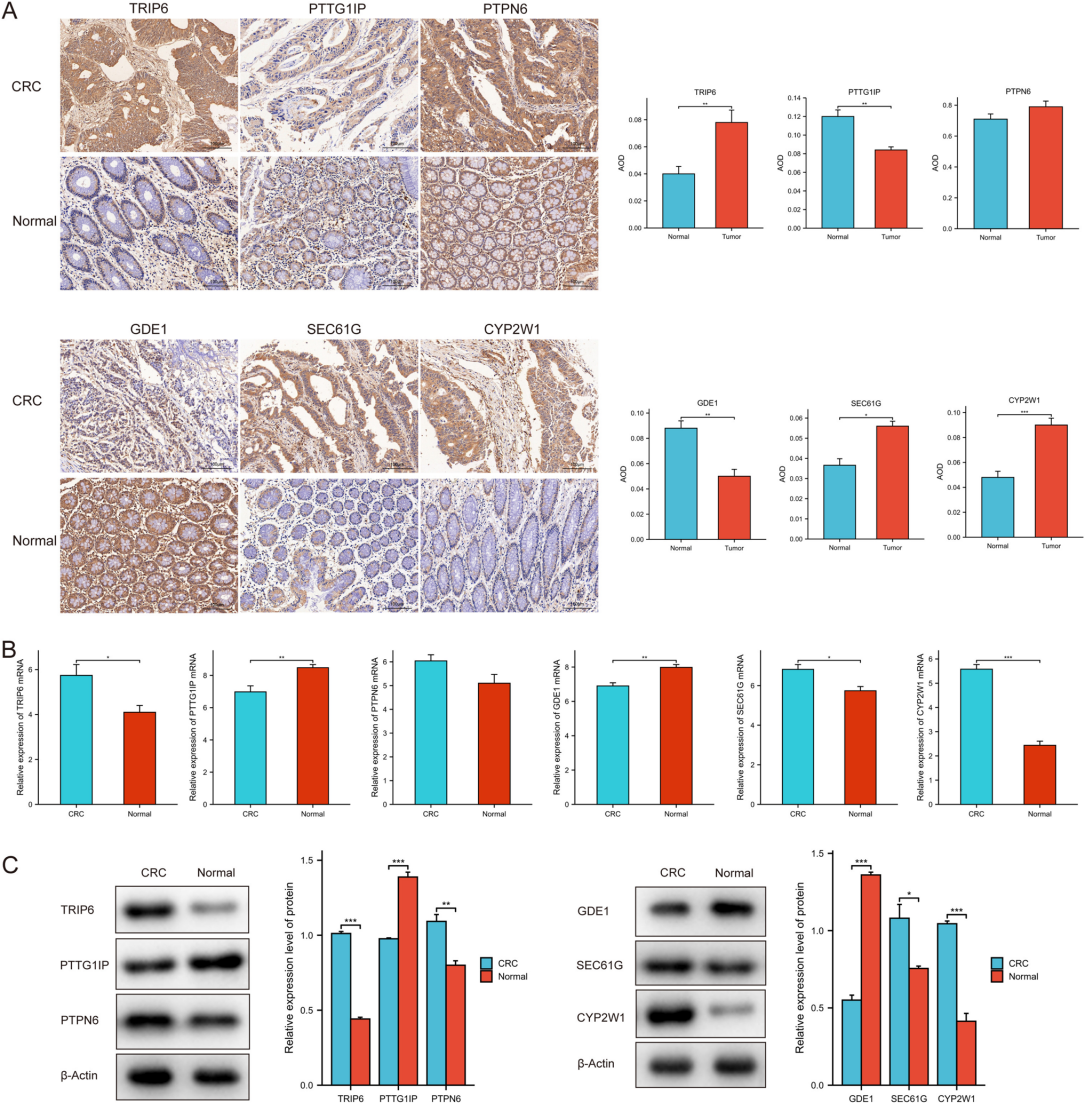
Validation of the expression levels of six key genes in CRC. **A** Representative images of IHC staining for six key genes in samples from patients with CRC. **B** RT‒qPCR analysis of the expression levels of six key genes in CRC tissues. **C** Western blotting analysis of the expression levels of six key genes in CRC samples. *P < 0.05, **P < 0.01, ***P < 0.001

We performed IHC, RT‒qPCR, and Western blotting to evaluate the relationship between CRC status and protein expression of the hub genes. As shown in Fig. 7A, PTTG1IP and GDE1 expression was reduced in CRC tissues compared to normal colon tissues, whereas *TRIP6, SEC61G*, and *CYP2W1* expression was markedly higher in CRC tissue samples than in normal colon tissue samples according to IHC. The mRNA expression levels of *TRIP6, SEC61G*, and *CYP2W1* were markedly higher in CRC tissues than in normal colon tissues according to RT‒qPCR analysis (Fig. 7B). Western blotting analysis demonstrated that the expression levels of *TRIP6, PTPN6*, *SEC61G,* and *CYP2W1* were considerably higher in CRC tissues than in normal colon tissues. Conversely, the expression level of GDE1 was significantly reduced in CRC tissues compared to normal colon tissues (Fig. 7C).

### Knockdown of TRIP6 inhibited CRC progression

**Fig. 8:**
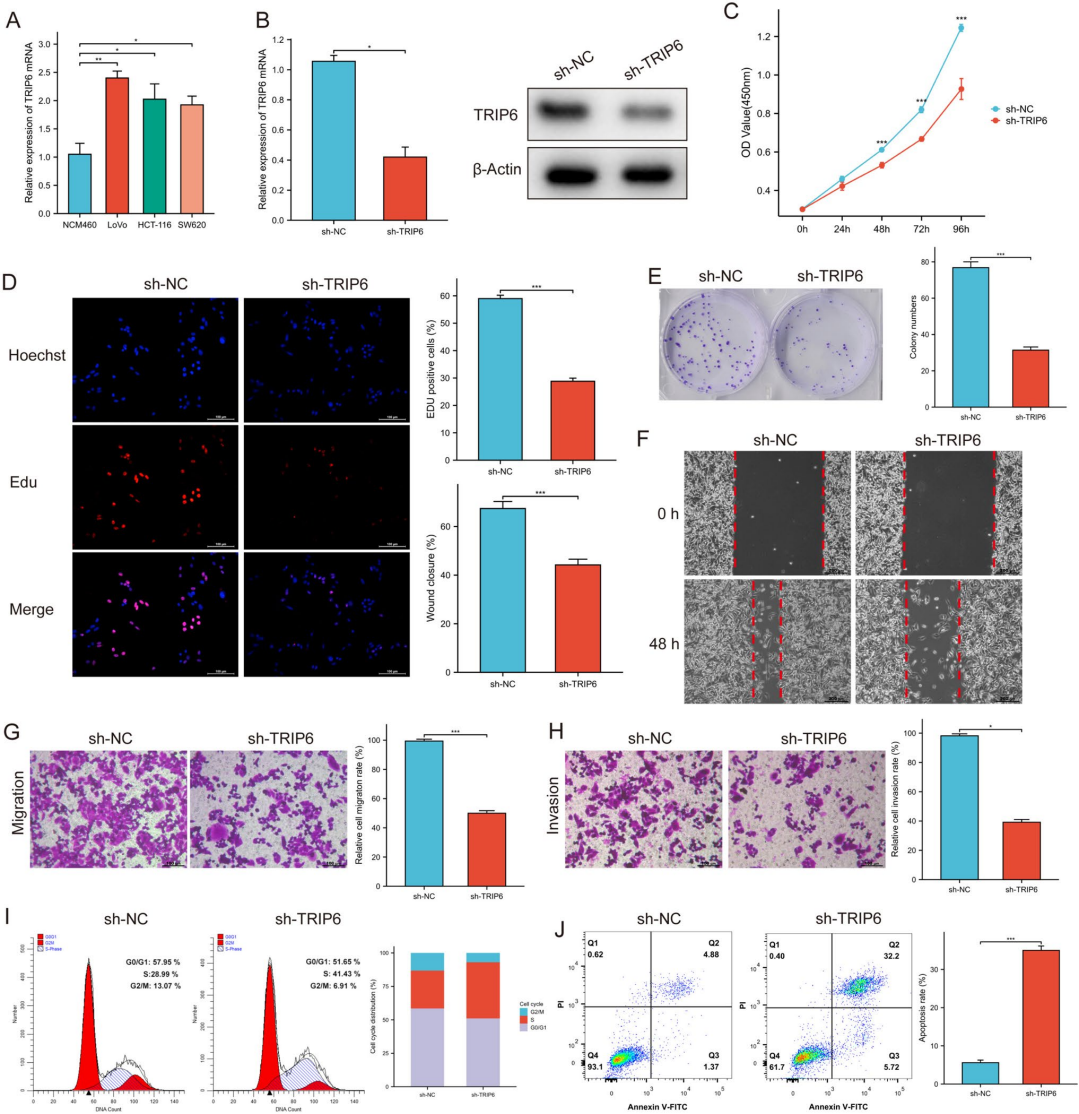
Silencing of TRIP6 inhibited CRC cell proliferation, invasion, and migration and promoted apoptosis. **A** TRIP6 expression was increased to various degrees in CRC cell lines and NCM460 cells. **B** Western blotting and RT‒qPCR analyses of TRIP6 expression in LoVo cells transfected with sh-TRIP6 or sh-RNA. **C** The cell proliferation rate after TRIP6 knockdown was determined via CCK-8 assays. **D** The effect of TRIP6 knockdown on LoVo cell proliferation was demonstrated via EdU assays. **E** Colony formation ability of TRIP6-knockdown cells. **F** Wound-healing assays showed decreased migration of LoVo cells transfected with sh-TRIP6. **G** The migration of TRIP6-knockdown cells was measured via Transwell assays. **H** The invasion of TRIP6-knockdown cells was assessed via Transwell assays. **I, J** Flow cytometric analysis and quantitative analysis showing cell cycle progression and cell death of LoVo cells transfected with sh-TRIP6. **P < 0.01, ***P < 0.001, ****P < 0.0001

To explore the biological roles of TRIP6, we studied the effects of *TRIP6* knockdown on a CRC cell line, namely, LoVo cells. We first validated the expression of TRIP6 in CRC cell lines via RT‒qPCR, and the LoVo cell line had the highest expression level of TRIP6 (Fig. 8A). RT‒qPCR and Western blotting analyses revealed a substantial decrease in the expression of TRIP6 in LoVo cells that were transfected with sh-TRIP6 (Fig. 8B). The CCK-8 assay showed that decreased TRIP6 expression slowed LoVo cell proliferation (Fig. 8C). The percentage of EdU-positive cells was lower in stable TRIP6-knockdown LoVo cells than in negative control cells (Fig. 8D).

In addition, the colony-forming capacities of LoVo cells were reduced in the TRIP6-knockdown group (Fig. 8E). The wound-healing assay showed that LoVo cells in the TRIP6-knockdown group traveled a considerably shorter distance than did those in the control group (Fig. 8F). Transwell assays revealed that knocking down TRIP6 resulted in a decrease in the migratory and invasive capabilities of LoVo cells (Fig. 8G, H). Compared with control cells, there were more TRIP6-knockdown LoVo cells in the S phase and fewer TRIP6-knockdown LoVo cells in the G2/M phase (Fig. 8I), and the proportion of apoptotic cells was markedly higher in the TRIP6-knockdown group (Fig. 8J).

## Discussion

The prognostic value of the heterogeneous and aggressive characteristics of CRC remains limited [59]. It is crucial to evaluate innovative risk models or subtype-specific risk variables to assist in the development of patient-specific treatments and improve patient prognosis [60]. scRNA-seq is used to characterize distinct cell populations and identify unique biomarkers and variations within different cell types in many cancers, including CRC [61, 62]. Using scRNA-seq, Poonpanichakul et al. discovered that cancer subpopulations with significant heterogeneity exhibit distinct responses to a specific chemotherapy [63]. In contrast, bulk RNA-seq reveals mean gene expression across all cells [64]. To construct a novel risk model, we performed an exhaustive analysis of bulk RNA-seq and scRNA-seq data in this study that demonstrates good efficacy in predicting prognosis and determining the response to immunotherapy in patients with CRC.

Zheng et al. verified a nine-gene profile associated with cancer-associated fibroblasts (CAFs) that may be used as a standalone predictive marker for patients with CRC who might not benefit from immunotherapy, but this study did not consider intercellular communication [65]. The heterogeneity of tumor samples also results in increased variation in cellular communication, and heterogeneity of interactions with the TME is essential for tumorigenesis and resistance to therapy [66]. Our results suggest that intercellular communication is an important mechanism among cell subtypes. GO and KEGG analyses of DEGs that were obtained from TCGA revealed significant enrichment of genes related to the cell cycle, EMT, and KRAS signaling pathways, which contribute to the development of CRC. Alterations in cyclins and EMT-related genes are common in CRC, and therapeutic intervention that targets aberrant cell cycle regulators may be advantageous in the treatment of CRC [67]. Multiple studies have confirmed that KRAS-related signaling pathways play crucial roles in CRC development [68].

The high-risk group showed significant enrichment in immunological processes. Thus, we hypothesized that the risk score could function as a possible prognostic marker for patients with CRC who are receiving immunotherapy. We evaluated several aspects of TMB, immune infiltration, and immune checkpoints. The results showed that the expression of various immune factors, including immunomodulators, chemokines, and cell receptors, significantly differed between the high- and low-risk groups, suggesting that patients with different risk scores may exhibit differences in their responses to and ability to benefit from targeted immunotherapy. Furthermore, the results of correlations between the model and clinicopathological features demonstrated that the model was strongly correlated with lymph node metastasis and tumor stage, suggesting that the predictive capability of the model also predicts overall survival.

Furthermore, druggable targets in patients with CRC and their corresponding drugs in the GDSC database were discovered using our prognostic models. The aforementioned findings supported the DEG enrichment outcomes. The high-risk group demonstrated greater drug resistance to gemcitabine, doxorubicin, mitomycin C, and bleomycin, whereas the low-risk group demonstrated significantly reduced drug resistance to docetaxel and metformin, indicating that people with low risk scores may be more likely to benefit from chemotherapy.

The six prognosis-related genes, *TRIP6, PTTG1IP, PTPN6, GDE1, SEC61G*, and *CYP2W1,* were screened using RSF analysis. We found that *TRIP6, PTPN6*, and *GDE1* were independent prognostic factors for OS of CRC patients. Zhang et al. established subcategories for CRC based on the cGAS-STING pathway. These subcategories may be utilized to predict patient prognosis using 27 DEGs but without identifying DEG-associated ceRNAs [69]. The regulatory networks involved play crucial roles in CRC progression. Therefore, we developed a ceRNA regulatory network to elucidate the potential mechanisms underlying CRC using six crucial DEGs. This approach can provide insights into the unidentified regulatory networks involved in CRC. In addition, based on the TCGA analysis, TRIP6 was highly expressed in CRC, and its high expression reduced the overall survival of CRC patients. Therefore, we performed further experiments focusing on *TRIP6*.

The patterns of gene expression and heterogeneity identified through bulk RNA-seq and scRNA-seq have often not been validated experimentally [70]. We first examined and confirmed to be highly expressed in CRC tissues by immunohistochemistry, RT-qPCR, and western blotting. *TRIP6* encodes a protein with three LIM (Lin-11, Isl-1, and Mec-3) zinc-binding domains [71]. TRIP6 activates Akt signaling to facilitate CRC drug resistance by directly interacting with PARD3 [72]. Moreover, by targeting TRIP6, miR-7 inhibits the proliferation and migration of CRC cells [73]. However, the investigation of TRIP6's role in CRC remains unexplored. In this study, the inhibition of TRIP6 in LoVo cells led to a substantial inhibition of cell proliferation and growth, induction of cell cycle arrest, and facilitation of tumor cell apoptosis. This suggests that TRIP6 induces apoptosis and inhibits the cell cycle, thereby promoting cell survival. Molecular network analysis suggested that TRIP6 expression levels were correlated with that of some oncogenes and pathways related to carcinogenesis in CRC (Fig. 6C, D). For example, the inhibition of liver metastasis in colon cancer is significantly observed upon suppression of the oncogene AKT1. Additionally, epithelial-to-mesenchymal transition (EMT) is a pivotal factor in facilitating CRC invasion and metastasis. Tumor metastasis is the result of cancer cell migration and invasion. Our results indicated the inhibitory effects of TRIP6 silencing on LoVo cell invasion and migration in vitro. Therefore, TRIP6 may be a possible therapeutic target for CRC treatment.

## Conclusions

In conclusion, our research exhaustively characterized the various cell subpopulations found in the colorectal cancer tissues. Key molecular mechanisms and potential therapeutic implications of targeting immune modifications in colorectal cancer were identified in our study. Moreover, *TRIP6* expression is upregulated in CRC. *TRIP6* promotes cell proliferation, migration, invasion, cell cycle dysregulation, inhibition of apoptosis, which may serve as a prognostic indicator for CRC. Clearly, further exploration of the use of *TRIP6* as a therapeutic target for CRC is warranted.

### Supplementary Information

Below is the link to the electronic supplementary material.

Supplementary file1 (PDF 20 kb)

Supplementary file2 (DOCX 13 kb)

## Data Availability

All data generated or analyzed during this study are included in this published article and its supplementary information files.

## Abbreviations

ANOVA: Analysis of variance
AUC: Area under the curve
CCK-8: Cell Counting Kit-8
ceRNA: Competitive endogenous RNA
COAD: Colon adenocarcinoma
CRC: Colorectal cancer
DEG: Differentially expressed gene
EMT: Epithelial–mesenchymal transition
GDSC: Genomics of Drug Sensitivity in Cancer
GEO: Gene Expression Omnibus
GO: Gene Ontology
GSEA: Gene set enrichment analysis
GSVA: Gene set-variant analysis
HMDD: MiRNA-associated disease database
IC_50_: Half-maximal inhibitory concentration
IHC: Immunohistochemistry
KEGG: Kyoto Encyclopedia of Genes and Genomes
KM: Kaplan–Meier
LASSO: Least absolute shrinkage and selection operator
miRNA: MicroRNA
NK: Natural killer
OS: Overall survival
PC: Principal component
PCA: Principal component analysis
READ: Rectal adenocarcinoma
ROC: Receiver operating characteristic
RSF: Random survival forest
scRNA-seq: Single-cell RNA sequencing
TCGA: The Cancer Genome Atlas
TMB: Tumor mutation burden
