# Supplementary figures and images for: Construction of an immune predictive model and identification of TRIP6 as a prognostic marker and therapeutic target of CRC by integration of single-cell and bulk RNA-seq data
Source: Cancer Immunol Immunother. 2024 Mar 2;73(4):69. doi: 10.1007/s00262-024-03658-w (PMC10908634; doi:10.1007/s00262-024-03658-w)

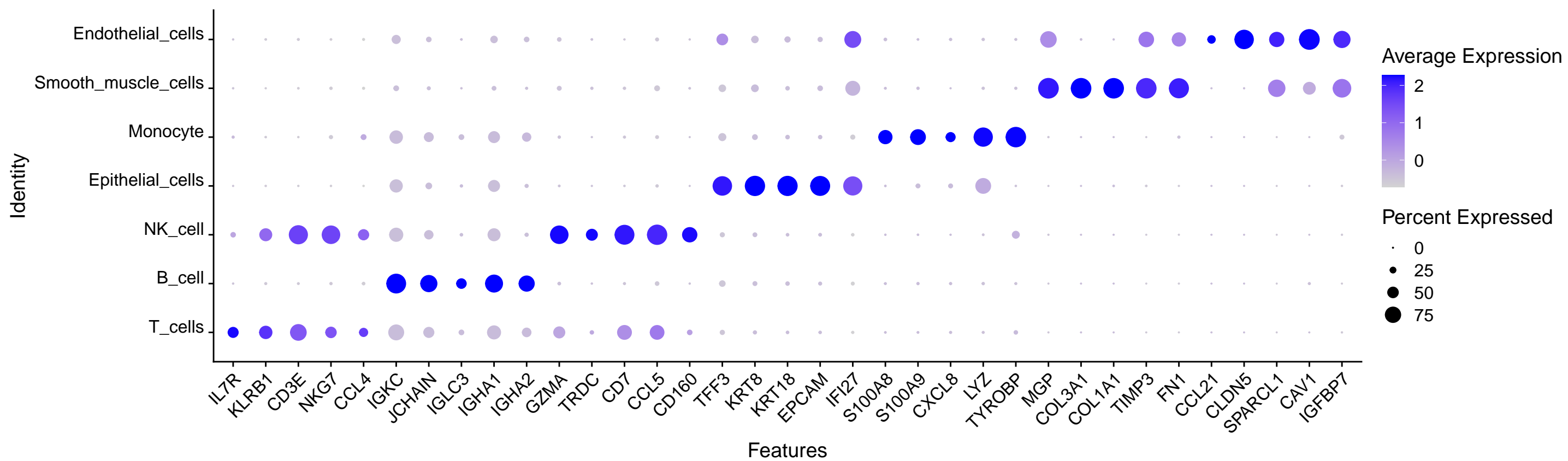

Supplement: Supplementary file 1 — Supplementary file1 (PDF 20 kb) [file 262_2024_3658_MOESM1_ESM.pdf]
