# Supplementary material for: Construction of an immune predictive model and identification of TRIP6 as a prognostic marker and therapeutic target of CRC by integration of single-cell and bulk RNA-seq data
Source: Cancer Immunol Immunother. 2024 Mar 2;73(4):69. doi: 10.1007/s00262-024-03658-w (PMC10908634; doi:10.1007/s00262-024-03658-w)
Supplement: Supplementary file 2 — Supplementary file2 (DOCX 13 kb) [file 262_2024_3658_MOESM2_ESM.docx]

**Table S1** RT-qPCR primer sequences

| **Gene** | **Forward Primer 5’-3’** | **Reverse Primer 5’-3’** |
| --- | --- | --- |
| TRIP6 | CAGATCCACTGCATTGAGGAC | CGCACTTGTAACAGCCAATG |
| PTTG1IP | GTCCTCTCGTCTTGCAGACTG | GCGGCTTCGTGTGCAGTTAG |
| GDE1 | ATGCACACAAGGCTACTGAG | TAGGCTCCAAGGTCTGTGAG |
| SEC61G | AGATGCACTAAACCTGATAG | CTTGTTCACCAATCTCTAAG |
| PTPN6 | GGAGGATGTGTATGAGAACC | ACCTGAGGACAGCACC |
| CYP2W1 | GAGCTCTTCCTGCTGTTTGC | GGCCTCATGGTAAAAGCCCG |
| β-actin | TGGACTCTGTTCGCTCAGGT | TGCCTCCTTCCGTACCACAT |
